# rDNA copy number variation and methylation from birth to sexual maturity

**DOI:** 10.18632/aging.206271

**Published:** 2025-06-16

**Authors:** Alina Michler, Sarah Kießling, Jana Durackova, Ramya Potabattula, Asuman Koparir, Thomas Haaf

**Affiliations:** 1 Institute of Human Genetics, Julius Maximilians University, 97074 Würzburg, Germany

**Keywords:** absolute rDNA copy number, active rDNA copy number, deep bisulfite sequencing, developmental delay, droplet digital PCR

## Abstract

Ribosomal DNA transcription is essential for ribosome biogenesis and the production of proteins. Using a combination of droplet digital PCR and deep bisulfite sequencing, we have quantified both the absolute number as well as the methylation level of individual rDNA transcription units (TU) in blood samples of 139 young healthy individuals and 141 sex- and age-matched individuals with unsolved syndromal developmental delay (DD), ranging from 0.02 to 18.4 years in age. There were no between-group differences in average promoter methylation, absolute copy number (CN), extreme CN, and hypomethylated (0-10%) presumably active CN. This largely excludes rDNA CN as a modulating factor in DD. The absolute CN in all 280 samples was 423.7 ± 108.4 (median 410, range 153 to 1,000) and the active CN was 175.0 ± 36.4 (median 174, range 70 to 376). Similar to adults, the absolute CN did not change from birth to sexual maturity but was strongly (Pearson *ρ* = 0.64, *P* < 0.001) correlated with promoter methylation. In contrast to adults, there was no significant correlation between age and promoter methylation and no age-related loss of active copies from birth to < 20 years. The number of completely unmethylated copies even significantly (Pearson *ρ* = 0.15; *P* = 0.01) increased during childhood and adolescence. Our results suggest that rDNA promoter methylation and the age-related loss of active rDNA TU, which are a hallmark of the aging process, start only after reaching sexual maturity.

## INTRODUCTION

The diploid human genome is endowed with several hundred ribosomal DNA (rDNA) transcription units (TU), which are arranged in tandem arrays in the short arms of the acrocentric chromosomes [1–3]. The encoded rRNA is essential for ribosome biogenesis in the nucleolus. Each cell must be fitted with an appropriate amount of ribosomes for messenger RNA translation and protein synthesis [4]. There is a complex interplay between rDNA methylation and epigenetic silencing [5–7]. Changes in rDNA methylation and transcription have been associated with development, aging and various age-related complex diseases [8, 9].

Previously, we have combined droplet digital PCR (ddPCR) and deep bisulfite sequencing (DBS) to count not only the absolute rDNA CN but also the CN with a given methylation value (i.e. 0%, 1-10%, etc.) [3, 10]. Consistent with the literature [1, 2], we found that the absolute CN was highly variable both in somatic tissue (blood) (mean ± SD 469 ± 107; median 469, range 243-895) and in haploid male germ cells (219 ± 47; median 214, range 98-404). Promoter hypomethylation is a prerequisite for rDNA transcription. In this light, we considered rDNA TU with 0-10% promoter methylation as likely active and TU with > 10% methylation as inactive. Using this classification system, the number of active rDNA TU was much smaller (182 ± 35; median 180, range 94-277 in blood and 108.7 ± 28.3, median 108, range 43-191 in sperm) than absolute CN [3, 10]. Notably, active CN did not depend on absolute CN, whereas the number of inactive copies increased with absolute CN. In both blood (donor age 15-71 years) and sperm (29-72 years), active rDNA CN decreased with age.

Although in the field of geroscience, the onset of the aging process is controversially discussed [11], it seems plausible to assume that aging begins after reaching sexual maturity [12, 13]. Numerous studies [3, 10, 14–18] using different techniques, species and tissues (including male and female germ cells) have shown a positive correlation between rDNA methylation and age. However, so far it is not known when the rDNA TU start to accumulate age-related CpG methylation errors. Most studies are based on linear regression models derived from data sets, representing adult and old age and extrapolating what happens before sexual maturity. Here we have analyzed absolute and active CN variation in two cohorts representing healthy controls and individuals with developmental delay (DD), respectively, in the age range from birth to < 20 years.

## RESULTS

Using DBS, we have analyzed mean methylation of the upstream control element and core promoter (UCE/CP) region in 139 samples from healthy individuals (control cohort) and 141 individuals with syndromal developmental delay (DD cohort). Age ranged from 0.07 to 18.4 (6.5 ± 4.4) years in the control and from 0.02 to 18.4 (6.4 ± 4.4) years in the DD cohort. There were no differences in UCE/CP methylation between the control (25.2 ± 9.1%, median 24.3%, range 3.6-47.8%) and the DD cohort (25.8 ± 8.6%, median 24.8%, range 8.2-50.2%) (Supplementary Figure 1). The absolute rDNA CN was determined by ddPCR. Consistent with our previous studies [3, 10], rDNA TU with 0-10% promoter methylation were classified as hypomethylated and presumably active. Both absolute CN (controls: 436.4 ± 125.2, median 424, range 153-1000; DD: 411.1 ± 87.4, median 406, range 248-877) and active CN (controls: 180.9 ± 41.8, median 174, range 86-376; DD: 169.1 ± 29.1, median 172, range 70-278) did not differ significantly between groups (Supplementary Figure 1). Moreover, the DD cohort was not enriched with extreme absolute or active CN. Neither the control (Pearson *ρ* = 0.06, *P* = 0.52) nor the DD cohort (*ρ* = -0.17, *P* = 0.05) showed a significant correlation between promoter methylation and age.

**Figure 1:**
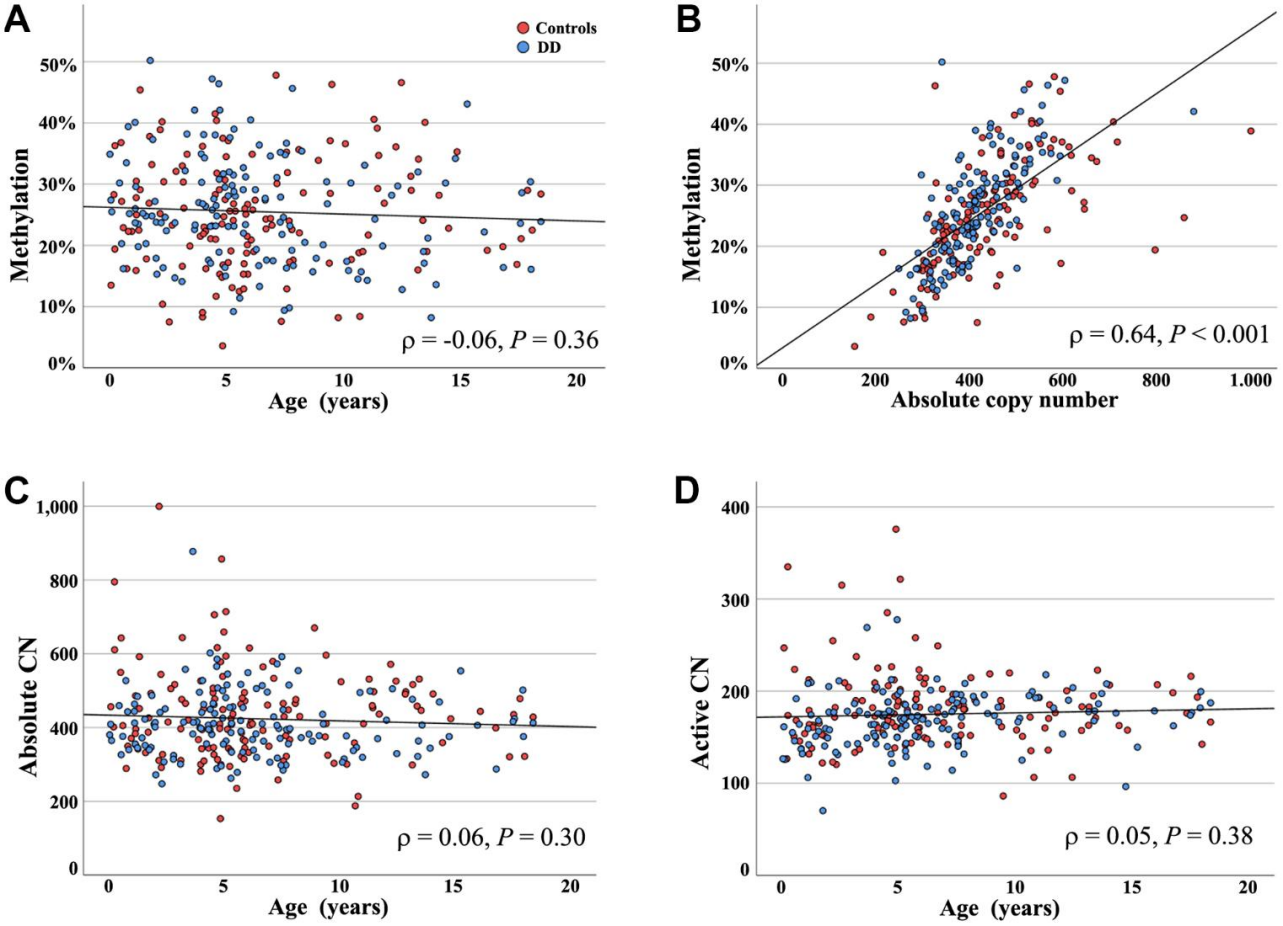
**UCE/CP methylation, absolute, and active CN in young individuals.** (**A**) Mean methylation (Y axis) of the UCE/CP does not increase with age (X axis). Red dots represent blood samples of 139 healthy controls and blue dots of 141 individuals with DD. (**B**) UCE/CP methylation (Y axis) is significantly positively correlated with absolute rDNA CN (X axis). (**C**, **D**) Both the absolute number of rDNA TU (**C**) and active CN (**D**) remain stable during the first 20 years of life. There are no significant between-group differences.

Because there were no between-group differences in CN variation and methylation both cohorts were combined for further analyses. In contrast to the well-known age effect in adults, there was no significant correlation (Pearson *ρ* = -0.06, *P* = 0.36) of UCE/CP methylation with age in the 280 young (< 20 years) individuals (Figure 1A). Similar to adults, promoter methylation showed a strong positive correlation (Pearson *ρ* = 0.64, *P* < 0.001) with the absolute CN (Figure 1B).

**Figure 2:**
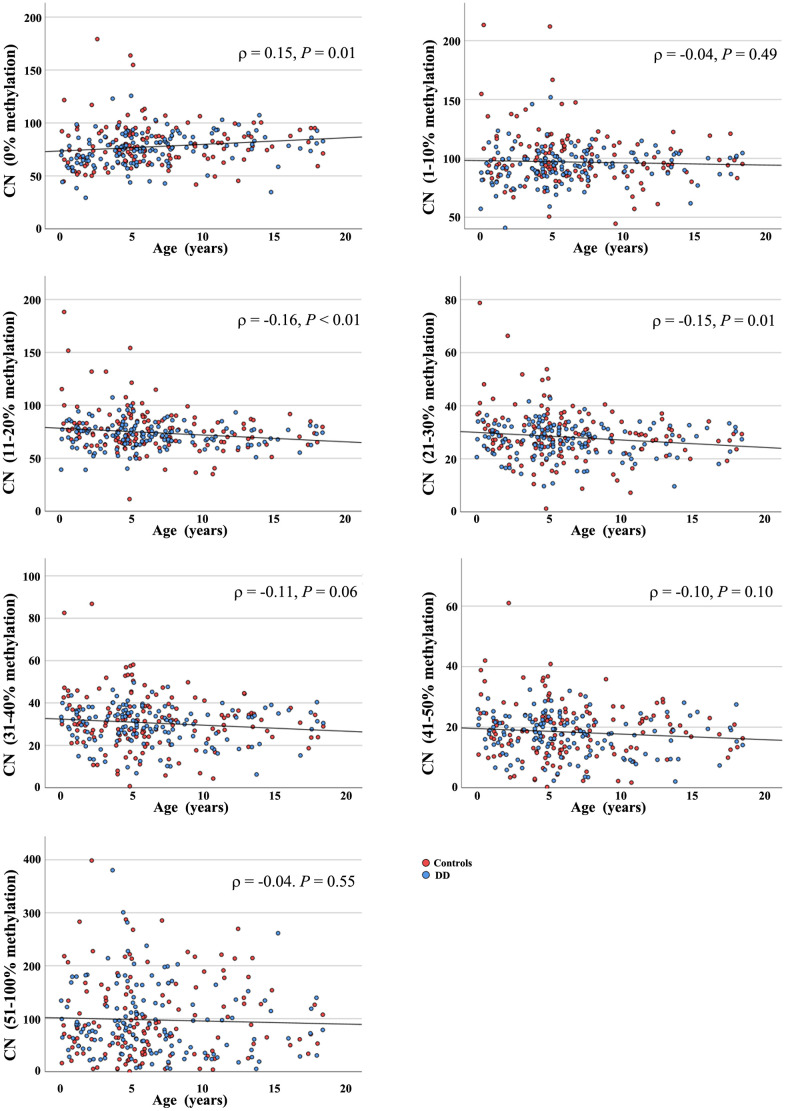
**Age-related methylation changes of the rDNA UCE/CP in young individuals.** The 139 healthy controls are indicated by red dots and 141 individuals with DD by blue dots. The Y axis shows the number of rDNA TU within a given methylation bin, the X axis indicates donor age, ranging from birth to < 20 years. The number of completely unmethylated (0%) promoter regions significantly increases with age, whereas the CN in the 1-10%, 11-20%, 21-30%, 31-40%, 41-50%, and 51-100% methylation bins is decreasing.

Next we classified rDNA copies into different methylation bins: 0%, 1-10%, 11-20%, 21-30%, 31-40%, 41-50%, and 51-100%. In both patients and controls 75-80 copies were completely unmethylated (controls: 79.8 ± 20.6, median 78; DD: 75.5 ± 15.7, median 76) and 90-100 (controls: 100.1 ± 23.5, median 95; DD: 93.7 ± 15.0, median 94) were lowly (1-10%) methylated (Supplementary Figure 2). When correlating the number of rDNA CN in a given methylation bin with age, the number of completely unmethylated TU in the 280 young individuals was significantly (Pearson *ρ* = 0.15; *P* = 0.01) increasing with age (from birth to < 20 years) (Figure 2). In all other methylation bins, 1-10% (*ρ* = -0.04; *P* = 0.49), 11-20% (*ρ* = -0.16; *P* < 0.01), 21-30% (*ρ* = -0.15; *P* = 0.01), 31-40% (*ρ* = -0.11; *P* = 0.06), 41-50% (*ρ* = -0.10; *P* = 0.10), and 51-100% (*ρ* = -0.04; *P* = 0.55) the number of copies were slightly decreasing with age. Although not all these correlations were significant, there was a clear trend towards gain of unmethylated (0%) and loss of hypermethylated (10-100%) copies from birth to sexual maturity.

The absolute CN in our young (birth to < 20 years) cohort ranged from 153 to 1,000 (423.7 ± 108.4; median 410) and the hypomethylated (0-10%) presumably active CN from 70 to 376 (175.0 ± 36.4; median 174). Neither absolute CN (Pearson *ρ* = -0.06; *P* = 0.30) (Figure 1C) nor active CN (*ρ* = 0.05; *P* = 0.38) (Figure 1D) changed from birth to < 20 years.

## DISCUSSION

### The age-related gain of rDNA methylation starts at sexual maturity

Previously we have shown that after sexual maturity aging of both the soma (from 15 to 71 years) and the male germline (from 29 to 72 years) was associated with increasing UCE/CP methylation, due to the loss of hypomethylated (0-10%) and gain of hypermethylated (11-100%) copies [3, 10]. Here we determined absolute CN and hypomethylated presumably active CN in 280 blood samples from young (birth to < 20 years) individuals. In both adult and young blood donors, absolute CN remained constant across the years. However, the age-related rDNA methylation dynamics differed between the two groups. In contrast to adult somatic tissue and sperm, there was no age-related loss of hypomethylated (0-10%) rDNA TU or gain of promoter methylation during young (< 20 years) age.

Collectively our data suggest that the rDNA hypomethylation state is actively maintained in somatic tissues of young individuals. The number of hypomethylated presumably active rDNA TU is even slightly increasing from birth to the beginning of the reproductive phase. An age-related gain of rDNA methylation in both soma and germline [3, 10] starts around 18-20 years of life. This is consistent with the idea that aging begins at sexual maturity [12, 13]. According to the disposable soma theory of aging [19], there is no need to regenerate the body after reproduction. Although speculative, we propose that following sexual maturity, there is no longer evolutionary pressure to prevent the accumulation of single CpG methylation errors, resulting in a loss of active rDNA copies.

### rDNA CN in individuals with developmental delay

Although ribosomes and, consequently rDNA CN and activity are involved in essentially all cellular processes, our knowledge on the role of CN variation in human health and disease is still limited. It has been proposed that rDNA CN may act as a modifier of multifactorial disease [1]. Very low and very high CN appear to be underrepresented in individuals older than 72 years and, therefore, may be disadvantageous for healthy aging [20]. In adult humans, rDNA CN has been associated with body mass index [21]. In addition, high CN have been associated with an increased schizophrenia risk and severity [22].

Ribosome dysfunction due to germline mutations in ribosomal protein-coding genes has been linked to various congenital disorders [23]. It is plausible to assume that rDNA CN may also be critical for normal development. Therefore, we have analyzed CN in a diverse spectrum of individuals with DD who remained unsolved after extensive genetic diagnostics (including chromosome banding, array CGH, and exome analysis). Individuals with DD showed the same CN and methylation variation as controls. In particular, we did not observe a higher number of individuals with extreme absolute and/or active CN in the DD group. Collectively, our results argue against the hypothesis that abnormal rDNA CN contributes to or modulates developmental delay, although we cannot exclude rare effects.

The repetitive nature and tandem arrangement of rDNA TU makes them susceptible to homologous recombination errors, resulting in enormous CN variation [24], which may affect cell functions and phenotypic traits. It seems plausible to assume that rDNA methylation is a mechanism which can compensate for the functional effects of extreme CN gains. There is a strong positive correlation between the absolute CN and the number of hypermethylated (> 10%) copies, which are epigenetically silenced [3, 10]. This ensures comparable active CN and rRNA activity in individuals with low and high absolute CN, respectively.

### Limitations

Overall, only a proportion (usually 30-60%) of all rDNA genes are active and unnecessary copies are epigenetically silenced [5–7]. Despite the complex interrelation between DNA methylation and gene expression, it is generally assumed that the amount of methylated CpGs in the promoter region plays an important role in the process determining whether a gene is active or not [25]. Previously we have shown that in both soma and germline the number of rDNA TU with no, one or two methylated CpGs (of the 25 analyzed CpGs) decreases during aging [3, 10], concluding that rDNA TU with 0-10% promoter methylation are active and copies with > 10% methylation inactive. However, so far functional experiments showing which CpG density turns a given TU on or off are missing. Moreover, the threshold for epigenetic silencing may vary between genes, cell types/tissues, and individuals.

Most studies including ours have analyzed rDNA CN in blood tissue, because it is easily accessible. Blood consists of different cell types, which can vary in cell composition between individuals. Active rDNA CN in blood cells may be primarily indicative of rDNA activity in the haematopoietic and the immune system, but does not necessarily represent the whole organism. On the other hand, accumulating evidence suggests that the intraindividual CN variation between different tissues is relatively small, compared to interindividual variation [21, 26]. Although this does not exclude that an individual tissue/organ in a particular patient may be endowed with extreme CN, compared to the other tissues (unpublished results), rDNA CN in blood may at least to some extent be representative of the soma.

## MATERIALS AND METHODS

### Study samples

The 139 healthy control samples were anonymized excess materials (blood DNA) from mutation-negative individuals in predictive diagnostics. Moreover, 141 individuals with DD, almost all of them with additional clinical symptoms (Supplementary Table 1) were included in this study. No underlying cause for DD was found by whole exome analysis. For most cases pathogenic CNVs and fragile X syndrome (in males) were also excluded. Samples of the control and the DD cohort were age- and sex-matched.

The DNA concentration and purity were determined using the Qubit dsDNA BR Assay system kit (Invitrogen, Karlsruhe, Germany). Bisulfite conversion was performed with the EpiTect Fast 96 Bisulfite kit (Qiagen, Hilden, Germany).

### Droplet digital PCR

ddPCR primers (Supplementary Table 2) for 28S rDNA and the human TATA-box binding protein (*TBP*) gene (internal reference) were adopted from Xue et al. (2017) [27]. ddPCR was performed exactly as described in our previous studies on rDNA CN variation [3, 10].

### Deep bisulfite sequencing

DBS was established according to the BisPCR2 protocol [28]. DBS primers (Supplementary Table 3) were designed for the human UCE/CP region. To reduce PCR errors, the first-round PCR primers have overhangs with the adapter sequences that are used to amplify the barcoded libraries in the second-round PCR. To minimize a PCR bias towards methylated or unmethylated DNA strands, the primers contain as few CpG sites as possible. For the single CpG near the 5´ end of the forward primer we used a degenerate Y (C or T) base to ensure unbiased amplification. The target region contained 25 contiguous CpGs and an A/G variant (GRCh37; chr13: 999,905) [3, 29]. For downstream analyses, only reads (> 95%) representing the major variant were considered.

First-round and second-round PCR reactions for DBS were performed, as described previously [3, 10]. The PCR cycling conditions were optimized by adjusting the annealing temperature. Higher annealing temperatures have been shown to improve amplification efficiency and reduce PCR bias [30]. In addition, we used a hot-start Taq DNA polymerase, which is known to reduce non-specific amplification. The purified and quantified PCR pools were combined into a single final pool for sequencing on the NextSeq 2000 platform (Illumina, CA, USA), as described previously [3, 10]. Since low-diversity libraries have a significant number of reads with identical sequence, which can shift the base composition, the rDNA sequencing library was spiked in with 20% PhiX, following the recommendations of Illumina. Adding such a high percentage of PhiX provided a good base diversity, cluster generation, and overall optimal run performance.

Sequencing with the Reagent Kit P1 (300 cycles) cartridge (Illumina) yielded 150 bp paired-end reads, which were processed using the Illumina BCL Convert software version 4.2.7 and analyzed further with Amplikyzer2 software [31]. The generated alignment files were thoroughly checked to ensure that reads containing poly-G sequences (resulting from the lower nucleotide diversity of the NextSeq2000) were filtered out.

### Statistical analysis

IBM SPSS software version 28 and R version 4.4.2. were used for statistical analyses. To determine the number of rDNA copies with a given methylation value the absolute CN (measured by ddPCR) was multiplied with the percentage of reads (measured by DBS) in the corresponding methylation bin.

## Supplementary Material

Supplementary Figures

Supplementary Table 1

Supplementary Tables 2 and 3
