# Supplementary material for: rDNA copy number variation and methylation from birth to sexual maturity
Source: Aging (Albany NY). 2025 Jun 16;17(6):1511–20. doi: 10.18632/aging.206271 (PMC12245198; doi:10.18632/aging.206271)
Supplement: Supplementary Figures [file aging-17-206271-s001.pdf]

SUPPLEMENTARY FIGURES

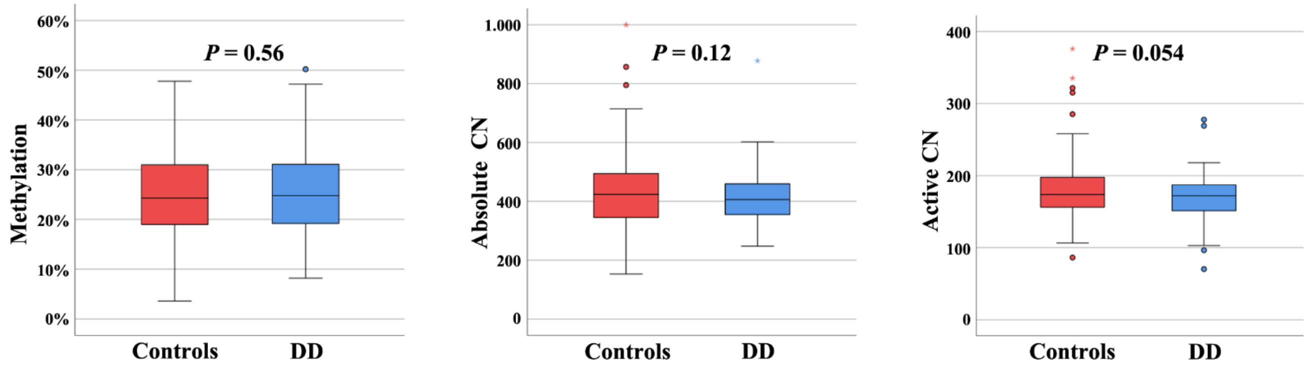

Supplementary Figure 1. UCE/CP methylation, absolute, and active CN in the control and the DD cohort. The left diagram shows the UCE/CP methylation in blood samples of 139 healthy controls (red boxes) and 141 individuals with DD (blue boxes). The middle diagram shows the absolute rDNA CN in control and DD individuals. The right diagram compares the number of hypomethylated (0-10%) active rDNA TU in both cohorts. The median is presented by a horizontal line. The bottom of the box indicates the 25th and the top the 75th percentile. Outliers are indicated by open circles and extreme outliers by stars. The Mann-Whitnex U test did not reveal significant between-group differences.

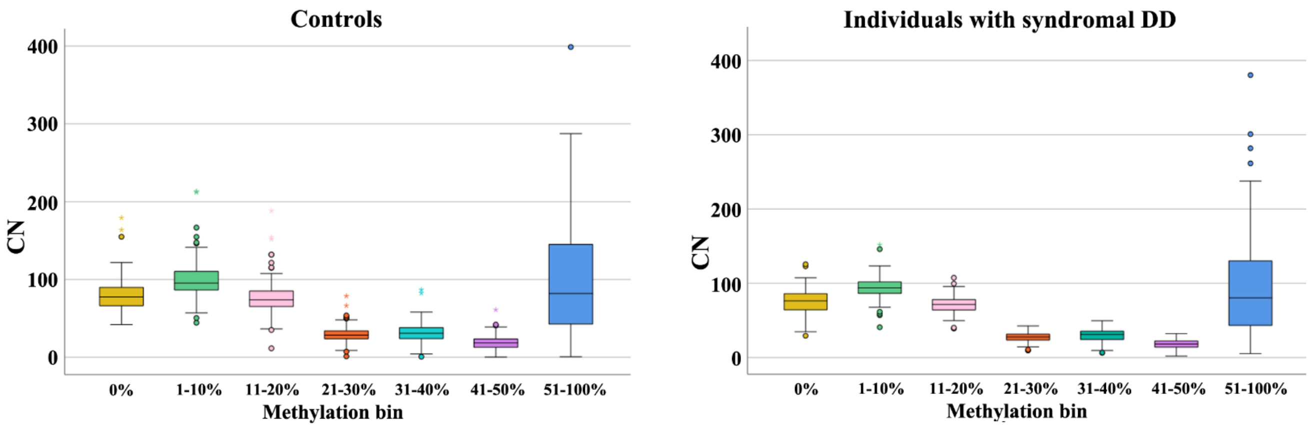

Supplementary Figure 2. Number and methylation distribution of rDNA (UCE/CP) copies in blood samples of 139 healthy controls and 141 individuals with DD. Box plots showing the number of rDNA copies (on the Y axis) within a given methylation range. Methylation bins representing 0%, 1-10%, 11-20%, 21-30%, 31-40%, 41-50%, and 51-100% are indicated on the X axis. The median is presented by a horizontal line. The bottom of the box indicates the 25th and the top the 75th percentile. Outliers are indicated by open circles, extreme outliers by stars. In controls 79.8 ± 20.6 (median 78) copies were completely (0%) and 100.1 ± 23.5 (median 95) lowly (1-10%) methylated. In individuals with syndromal DD 75.5 ± 15.7 (median 76) copies were completed and 93.7 ± 15.0 (median 94) lowly methylated.
