# Supplementary material for: rDNA copy number variation and methylation from birth to sexual maturity
Source: Aging (Albany NY). 2025 Jun 16;17(6):1511–20. doi: 10.18632/aging.206271 (PMC12245198; doi:10.18632/aging.206271)
Supplement: Supplementary Table 1 [file aging-17-206271-s002.docx]

**Supplementary Table 1.** Key clinical findings of DD probands.

| **ID** | **Age (years)** | **Sex** | **Symptoms*** |
| --- | --- | --- | --- |
| DD1 | 0.02 | female | multiple congenital abnormalities, NDD |
| DD2 | 0.06 | male | severe global NDD |
| DD3 | 0.10 | female | arthrogryposis multiplex congenita, brain abnormalities (cMRI) |
| DD4 | 0.43 | female | global NDD, short stature, seizures |
| DD5 | 0.52 | male | short stature, microcephaly, mild to moderate ID, submucosal cleft palate, scoliosis |
| DD6 | 0.59 | male | NDD, tall stature |
| DD7 | 0.72 | male | severe global NDD |
| DD8 | 0.80 | male | facial dysmorphism, microcephaly, short stature, DD |
| DD9 | 0.85 | female | global NDD, short stature, hypotonia |
| DD10 | 1.09 | male | short stature, facial dysmorphism, mild NDD |
| DD11 | 1.10 | male | hydrocephalus, global NDD, facial dysmorphism |
| DD12 | 1.10 |  | severe global NDD, brain abnormalities (cMRI) |
| DD13 | 1.13 | female | mild NDD, tall stature, hemihypertrophy |
| DD14 | 1.23 | male | moderate ID |
| DD15 | 1.42 | female | global NDD, brain abnormalities (cMRI) |
| DD16 | 1.55 |  | global NDD, hypotonia, short stature, facial dysmorphism |
| DD17 | 1.55 | female | global NDD, mild short stature, mild facial dysmorphism |
| DD18 | 1.73 | male | global NDD, facial dysmorphism |
| DD19 | 1.81 | male | short stature, NDD, recurrent infections |
| DD20 | 1.85 | male | speech delay, hydrocephalus, tall stature, macrocephaly, facial dysmorphism |
| DD21 | 1.94 | female | multiple congenital abnormalities, NDD |
| DD22 | 1.98 | female | global NDD, hearing impairment |
| DD23 | 1.99 | male | mild global NDD, iris coloboma |
| DD24 | 2.02 | male | global NDD, brain abnormalities (cMRI) |
| DD25 | 2.20 | male | global NDD, brain abnormalities (cMRI), seizures |
| DD26 | 2.28 | male | mild NDD, short stature |
| DD27 | 2.37 | male | speech impairment, motor developmental delay |
| DD28 | 2.42 | female | global NDD, microcephaly, seizures |
| DD29 | 2.78 | male | global NDD, pulmonary valve stenosis, microcephaly, ptosis |
| DD30 | 2.79 | female | clinical suspicion of Noonan syndrome |
| DD31 | 3.00 | female | speech impairment, ID |
| DD32 | 3.09 | male | speech impairment, motor developmental delay, mild facial dysmorphism |
| DD33 | 3.22 | male | global NDD, ASD |
| DD34 | 3.30 | male | global NDD |
| DD35 | 3.62 | male | ID |
| DD36 | 3.62 | male | global NDD |
| DD37 | 3.65 | female | speech impairment, seizures |
| DD38 | 3.80 | Male | speech impairment, mild motor developmental delay, ASD, behavioral abnormalities |
| DD39 | 3.91 | female | global NDD, mild facial dysmorphism |
| DD40 | 3.93 | male | tall stature, macrocephaly, NDD |
| DD41 | 3.97 | male | global NDD, seizures |
| DD42 | 4.01 | male | speech impairment, behavioral abnormalities |
| DD43 | 4.05 | female | speech impairment, behavioral abnormalities |
| DD44 | 4.09 | male | global NDD, behavioral abnormalities, seizures |
| DD45 | 4.22 | female | absent speech, strabismus, Pierre Robin sequence |
| DD46 | 4.30 | female | speech impairment, ASD/ADHD |
| DD47 | 4.31 | female | speech impairment, mild motor developmental delay, behavioral abnormalities, tethered cord, mild facial dysmorphism |
| DD48 | 4.36 | male | speech impairment, macrocephaly, facial dysmorphism |
| DD49 | 4.37 | female | ID, speech impairment |
| DD50 | 4.38 | male | ID, visual impairment |
| DD51 | 4.45 | male | speech impairment, motor developmental delay |
| DD52 | 4.50 | male | global NDD, macrocephaly |
| DD53 | 4.52 | male | speech impairment, ASD |
| DD54 | 4.54 | male | speech impairment |
| DD55 | 4.57 | male | speech impairment, ASD |
| DD56 | 4.66 | male | short stature, microcephaly, global NDD, visual impairment, facial dysmorphism |
| DD57 | 4.67 | male | global NDD, seizures, visual impairment |
| DD58 | 4.67 | male | speech delay, macrocephaly, 2-3 partial syndactyly of the toes |
| DD59 | 4.70 | male | speech impairment, motor developmental delay, ASD |
| DD60 | 4.85 | male | speech impairment, behavioral abnormalities |
| DD61 | 4.92 | male | Tetralogy of Fallot, ID, short stature |
| DD62 | 4.92 | male | speech impairment, behavioral abnormalities, mild facial dysmorphism |
| DD63 | 4.94 | female | ID, ASD, seizures |
| DD64 | 5.05 | male | speech impairment, motor developmental delay |
| DD65 | 5.06 | male | ID, speech impairment, motor developmental delay |
| DD66 | 5.10 | female | ID, ADHD, speech impairment |
| DD67 | 5.13 | male | global NDD, ataxia |
| DD68 | 5.22 | male | ID, speech impairment |
| DD69 | 5.28 | male | seizures, mild NDD |
| DD70 | 5.28 | female | mild NDD, short stature |
| DD71 | 5.30 | female | absent speech, motor developmental delay, mild facial dysmorphism |
| DD72 | 5.30 | male | speech delay, ASD, mild facial dysmorphism, preauricular tag |
| DD73 | 5.30 | male | speech impairment, behavioral abnormalities, short stature |
| DD74 | 5.36 | male | speech impairment, microcephaly, short stature |
| DD75 | 5.55 | male | ID |
| DD76 | 5.69 | male | global NDD |
| DD77 | 5.70 | mal | mild ID |
| DD78 | 5.71 | female | speech impairment, delayed fine and gross motor skills, visual impairment |
| DD79 | 5.79 | male | ID, ASD |
| DD80 | 5.80 | male | global NDD |
| DD81 | 6.01 | female | speech impairment, ASD |
| DD82 | 6.21 | female | speech delay, short stature, microcephaly |
| DD83 | 6.25 | male | mild ID, ataxia, motor developmental delay |
| DD84 | 6.36 | female | speech impairment, motor developmental delay, behavioral abnormalities |
| DD85 | 6.38 | female | speech impairment, motor developmental delay, mild facial dysmorphism |
| DD86 | 6.39 | female | septooptic dysplasia, ID |
| DD87 | 6.63 | female | global NDD, short stature |
| DD88 | 6.63 | female | global NDD, macrocephaly |
| DD89 | 6.69 | male | global NDD, ADHD |
| DD90 | 6.85 | male | ID, speech impairment, ASD? |
| DD91 | 6.97 | male | ID, speech impairment, ASD |
| DD92 | 7.13 | male | global ND, tall stature, strabismus, behavioral abnormalities |
| DD93 | 7.27 | male | ID |
| DD94 | 7.28 | female | ID, speech impairment, motor developmental delay, behavioral abnormalities |
| DD95 | 7.29 | female | moderate ID, scoliosis |
| DD96 | 7.35 | female | mild to moderate ID, reflex epilepsy, hypotonia, supraumbilical hernia |
| DD97 | 7.41 | female | ID, facial dysmorphism |
| DD98 | 7.45 | male | speech impairment, motor developmental delay, behavioral abnormalities |
| DD99 | 7.48 | male | mild ID, ADHD, short stature |
| DD100 | 7.54 | female | macrocephaly, global NDD (mild to moderate) |
| DD101 | 7.54 | male | mild ID, speech impairment, visual impairment, mild facial dysmorphism |
| DD102 | 7.66 | female | speech impairment, motor developmental delay |
| DD103 | 7.69 | male | mild ID, ADHD |
| DD104 | 7.77 | male | global NDD |
| DD105 | 7.79 | female | ID, mild facial dysmorphism |
| DD106 | 7.83 | male | mild ID, severe behavioral abnormalities |
| DD107 | 8.19 | male | global NDD, short stature, ID, strabismus, cerebellar hypoplasia (cMRI) |
| DD108 | 8.32 | male | global NDD, macrocephaly |
| DD109 | 8.66 | male | global NDD, short stature |
| DD110 | 8.80 | male | ID, behavioral abnormalities |
| DD111 | 9.10 | female | mild ID, speech impairment |
| DD112 | 9.25 | male | speech impairment, mild ID, multiple lentigines, hyperopia |
| DD113 | 9.28 | male | ID, ASD |
| DD114 | 10.11 | female | absent speech, facial dysmorphism, severe global NDD, ataxia |
| DD115 | 10.18 | male | speech delay, ID, ASD |
| DD116 | 10.28 | male | ID, speech impairment, macrocephaly |
| DD117 | 10.58 | male | mild ID, behavioral abnormalities, mild facial dysmorphism |
| DD118 | 10.72 | female | global NDD, obesity, facial dysmorphism, visual impairment |
| DD119 | 10.87 | male | ID, absent speech, ASD |
| DD120 | 10.98 | male | ID, behavioral abnormalities |
| DD121 | 11.30 | female | global NDD, facial dysmorphism |
| DD122 | 11.64 | male | speech impairment, motor developmental delay, tall stature, micropenis |
| DD123 | 12.02 | female | ID, ADHD |
| DD124 | 12.28 | female | ID |
| DD125 | 12.47 | male | speech impairment, motor developmental delay, behavioral abnormalities |
| DD126 | 13.14 | female | mild NDD, congenital heart defect |
| DD127 | 13.37 | male | ID, speech impairment, behavioral abnormalities |
| DD128 | 13.40 | male | mild ID, severe behavioral abnormalities |
| DD129 | 13.56 | female | moderate ID |
| DD130 | 13.70 | male | ID, visual impairment |
| DD131 | 13.91 | male | ID, speech impairment, behavioral abnormalities |
| DD132 | 14.31 | female | mild ID, mild motor developmental delay, behavioral abnormalities |
| DD133 | 14.74 | male | global NDD, ataxia |
| DD134 | 15.24 | female | ID, behavioral abnormalities |
| DD135 | 15.96 | female | ID |
| DD136 | 16.78 | male | ID, ADHD |
| DD137 | 17.51 | male | ID, ADHD |
| DD138 | 17.57 | male | absent speech, motor developmental delay, ASD |
| DD139 | 17.93 | male | ID, behavioral abnormalities, facial dysmorphism |
| DD140 | 17.96 | male | ID |
| DD141 | 18.38 | male | ID, visual impairment |

*Abbreviations: ID, intellectual disability; NDD, neurodevelopmental delay; ASD, autism spectrum disorder; ADHD, attention deficit hyperactivity disorder.
