# Supplementary material for: rDNA copy number variation and methylation from birth to sexual maturity
Source: Aging (Albany NY). 2025 Jun 16;17(6):1511–20. doi: 10.18632/aging.206271 (PMC12245198; doi:10.18632/aging.206271)
Supplement: Supplementary Tables 2 and 3 [file aging-17-206271-s003.pdf]

SUPPLEMENTARY TABLES

Supplementary Table 2. Primers for ddPCR of human rDNA.

Assay	Primer	Sequence (5’-3’)[a]
28S rDNA	Forward	5’-AACGTGAGCTGGGTTTAG-3’
	Reverse	5’-CTCGTACTGAGCAGGATTAC-3’
	Probe	5’-/5HEX/TGGCAACAA/ZEN/CACATCATCAGT/3IABkFQ/-3’
TBP	Forward	5’-GATATGAGACTGTGGGTAAGT-3’
	Reverse	5’-GATCCTTTGAACACCCTAATG-3’
	Probe	5’-/56-FAM/ACAGAGATC/ZEN/ACTGCAGTTGC/3IABkFQ/-3’

Supplementary Table 3. Primers for deep bisulfite sequencing of the human rDNA promoter (UCE/CP) region.

Primer	Sequence (5’-3’)[a]	Amplicon length	Variant	Annealing Temp. (° C)	No. of CpGs
Forward	TATTYGGAGGTTTAATTTTTTTAG	239 bp	A/G*	56ºC	25
Reverse	TATATCCTAAAATTAACCAAAAAACCCC				

* indicates the major allele.
